# Heterotopic pancreas and malakoplakia in abscessing intrahepatic tumor – a case report

**DOI:** 10.1515/iss-2025-0002

**Published:** 2025-03-28

**Authors:** Marc A. Reschke, Phil Meister, Sven A. Lang, Dirk Theegarten, Tom F. Ulmer, Hideo A. Baba, Ulf P. Neumann, Lara Heij, Sophia M. Schmitz

**Affiliations:** Department of General, Visceral, Vascular and Transplantation Surgery, 39081 University Hospital Essen, Hufelandstr. 55, 45147, Essen, Germany; Institute of Pathology, University Hospital Essen, Essen, Germany

**Keywords:** heterotopic pancreas, malakoplakia, intrahepatic tumor

## Abstract

**Objectives:**

The presence of heterotopic pancreatic tissue or malacoplakia in the liver is very uncommon and rarely becomes symptomatic.

**Case presentation:**

Here we report of a 57-year-old female patient who received left-sided hemihepatectomy due to a tumor resembling intrahepatic abscess. Histopathological examination revealed both heterotopic pancreas and malakoplakia.

**Conclusions:**

We discuss this case in relation to the existing literature. As far as we know, this is the first reported case of simultaneous presence of these two rare entities.

## Introduction

### Heterotopic pancreas

Heterotopic pancreas is defined as pancreatic tissue without anatomical or vascular relation to the pancreas. It most likely develops during the embryonal period [1].

It is mostly found in the gastrointestinal (GI) tract, especially in the stomach, duodenum, jejunum and Meckel’s diverticulum [2]. In the hepatobiliary system heterotopic pancreas occurs especially in the gallbladder as incidental finding after cholecystectomy [3]. However, pancreatic heterotopia in the liver itself has been rarely described.

Terada et al. [2] examined 1,000 consecutive autopsies for the presence of pancreatic heterotopia in the liver. They showed an incidence of intrahepatic pancreatic heterotopia of 4.1 % suggesting pancreas heterotopia in the liver is more common than actually expected.

Histopathologically it occurs in relation to large and medium sized intrahepatic ducts and secretes pancreatic enzymes into the bile duct [1], 2].

Although in literature pancreas heterotopia is generally described as asymptomatic, symptoms may occur particularly in the GI tract [4]. Nonetheless, due to the lack of symptoms, it is still unclear how often it occurs in the liver.

In very rare cases, these lesions can mimic biliary tract obstruction and Klatskin tumors. Only 10 cases have been reported on pancreas heterotopia as a reason for bile duct obstruction mimicking cholangiocarcinoma [5]. Yu et al. reported on a patient with hepatolithiasis requiring left hemihepactomy. In the histopathological analysis they incidentally found pancreas heterotopia suggesting pancreas heterotopia to be responsible for the development of hepatolithiasis [1]. There is only one case report in which the suspected development of intrahepatic adenocarcinoma on the ground of the heterotopia has been described [6].

### Malakoplakia

Malakoplakia is a rare inflammatory response due to lack of lysosomal function resulting in defective reaction of macrophages to phagocytosed bacteria [7]. It is mainly described in the genitourinary tract as well as in the gastrointestinal tract and cutaneous in association to immunosuppression, infections (tuberculosis, HIV), sarcoidosis and steroid use [8]. Macroscopically malakoplakia presents as soft white plaques [8] whereas histopathologically Michaelis–Gutmann bodies are pathognomonic [7]. The first case of malakoplakia was described in 1991. Since then less than 10 cases of malakoplakia in the liver have been published in the English literature [7]. Therapeutic approaches mentioned in existing case reports are limited to antibiotic therapy and surgical treatment.

## Case presentation

**Figure 1:**
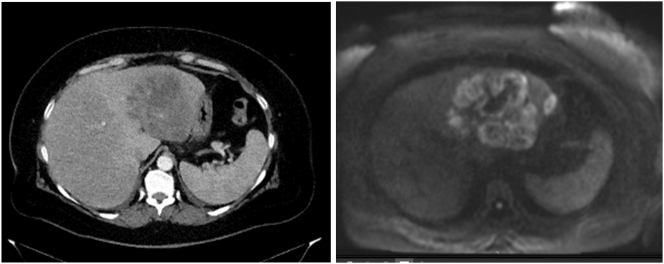
CT scan upon admission to the hospital (left) and MRI (right) showing a polylobulated, cystic, partly solid tumourous process of the left lobe of the liver.

We report a 57-years-old female patient referred to the hospital due to epigastric pain, fever, and worsening general condition that had been progressive for several days. Except for obesity grade II (WHO), there were no previous illnesses except of cholangitis after cholangiography and stent placement due to gallstones. In her history laparoscopic cholecystectomy was performed. Blood tests showed increased infection parameters and blood cultures revealed evidence for *Klebsiella pneumoniae*. Computer tomography (CT) and magnetic resonance imaging (MRI) (Figure 1) were performed showing a polylobulated, cystic, partly solid tumourous process of the left liver lobe. Tumor markers were normal. There were no signs for pulmonary metastases. Needle biopsy was performed without any histological evidence for malignancy. Endoscopic retrograde cholangiopancreatography revealed stenosis suspicious for malignancy and a stent was placed in the common bile duct.

**Figure 2:**
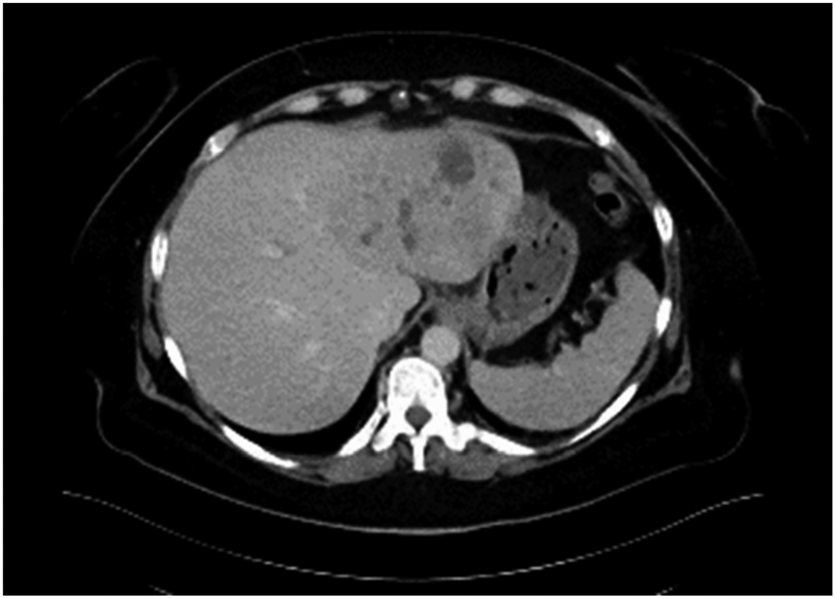
CT scan after antibiotic treatment and bile duct stenting preoperatively.

The patient was relocated to our center for further treatment. Gastroscopy and colonoscopy showed no sign of malignancy except of a colon polyp that was completely removed. In the follow-up CT scan (Figure 2) after antibiotic therapy the abscess formation was regressive with a remaining suspicious mass. Imaging was suggestive of an intratumoral abscess, and a left-sided hemihepatectomy was performed. The further postoperative course was overall without serious side events. After completion of antibiotic therapy due to *Enterococcus faecium* in the blood culture, the patient was discharged home 18 days after surgery in excellent clinical condition.

### Pathological examination

**Figure 3:**
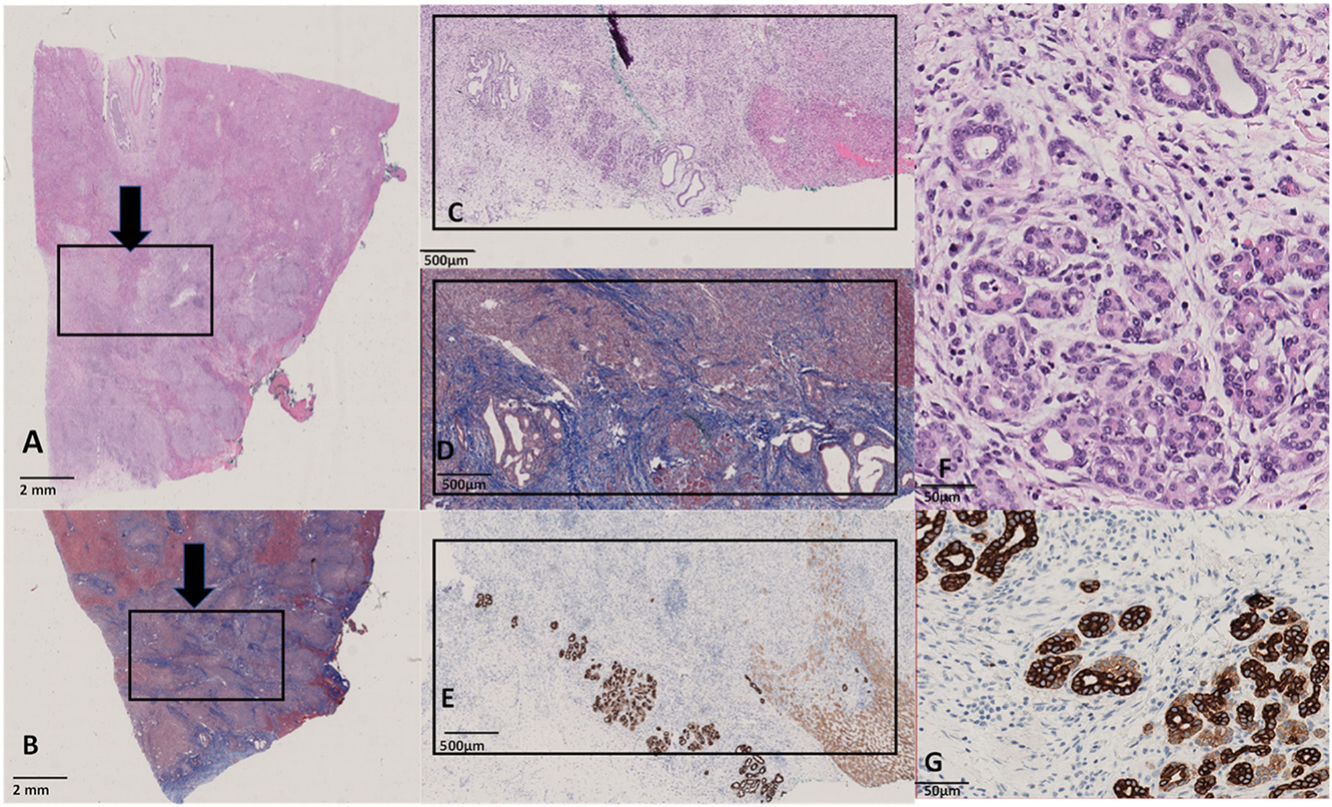
Histology of the pancreatic heterotopia in the liver. (A) Hematoxylin and eosin stain (HE) of the section demonstrating the intratumoral mass (black arrow). The black box indicates the region with pancreatic heterotopia, located at the edge of the lesion. (B) Masson Goldner’s (MGE) stain of the intratumoral lesion demonstrates the fibrotic nature of the lesion. (C) HE with a zoomed-in box of the pancreatic heterotopia. Here, we see the acinar ducts, as well as the pancreas parenchyma. (D) Zoomed-in box in the MGE stain, demonstrating the fibrosis. (E) Zoomed-in box in the Cytokeratin 7 (CK7) stain, here we see the epithelium stained in brown. At the right corner, the liver parenchyma demonstrates ductular metaplasia. The architecture of the pancreatic heterotopia is demonstrated. No cancerous lesions are present. (F) HE with a zoomed-in box of the pancreatic heterotopia. Here, we see the acinar ducts, as well as the pancreas parenchyma. (G) Zoomed-in box in the Cytokeratin 7 (CK7) stain, here we see the epithelium stained in brown in the architecture of pancreas parenchyma.

**Figure 4:**
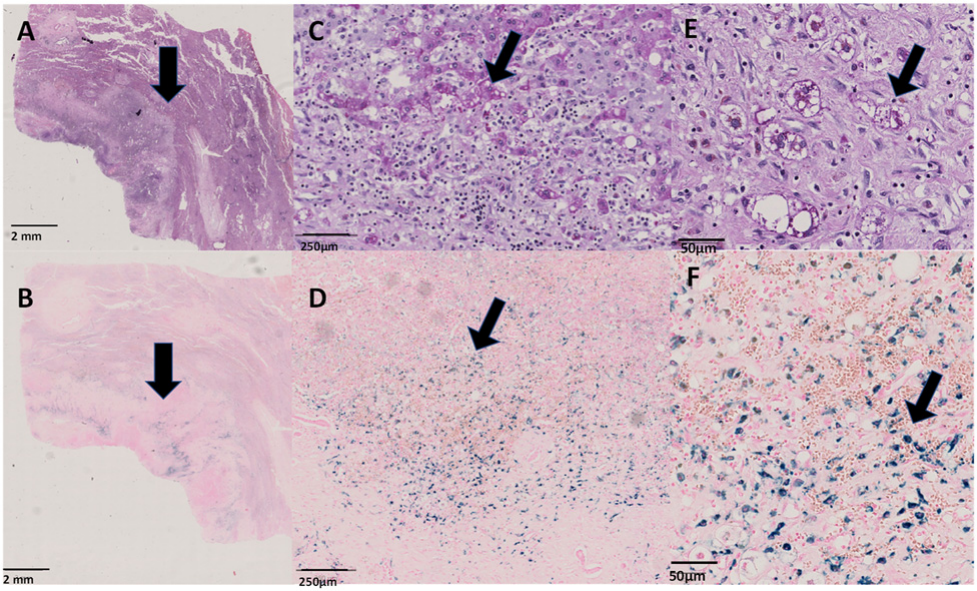
Histology of Malakoplakia in the liver. (A) HE stain of the section demonstrating the intratumoral mass (black arrow). The black arrow indicates the region with histiocytes surrounding the liver mass, located at the edge of the lesion. (B) Iron stain of the intratumoral lesion demonstrates positive iron in blue. (C) Zoomed-in box of the histiocytes in the PAS stain located at the edge of the lesion. The black arrow points to the positive globules in the histiocytes. (D) Zoomed-in image of the histiocytes in the iron stain located at the edge of the lesion. The black arrow points to the positive globules in the histiocytes in blue. (E) Zoomed-in image of the histiocytes in the PAS stain located at the edge of the lesion. The black arrow points to the positive globules in the histiocytes. (F) Zoomed-in image of the histiocytes in the iron stain located at the edge of the lesion. The black arrow points to the positive globules in the histiocytes in blue.

The specimen was examined at our pathology department and macroscopy revealed an intratumoral mass. The lesion was embedded and histology showed a fibrotic lesion with inflammation and heterotopic pancreatic tissue. The regions with pancreas parenchyma show the acinar ducts as well as the neuroendocrine Langerhans islands (Figure 3). Additionally, pathological examination showed the presence of malakoplakia (Figure 4).

## Discussion

In the presented case, both heterotopic pancreas and malakoplakia were mimicking an abscessing intrahepatic tumor. To our knowledge this is the first clinical and histological report of simultaneous presence of these two extremely rare entities (Figures 3 and 4).

As a common practice in hepatobiliary surgery, resection was carried out following guidelines, as there was a high degree of suspicion for malignancy. Heterotopic pancreas cannot be reliably identified or differentiated from an image morphological point of view [9]. Ultimately, the malakoplakia was also remedied, which represents a basic therapy recommendation in literature [8].

Because the patient had no history of immunospression or any other illnesses it is most likely that the abscessing tumor was caused due to chronic inflammation in the liver. The reason for that cannot be clarified with absolute certainty. Pancreatic enzymes present intrahepatically due to pancreatic heterotopia may cause direct destruction of the liver tissue. In addition, exocrine enzymes change the composition of the bile acid over time [1] which results in chronic inflammation. Chronic inflammation subsequently suppresses the immune response, making the tissue more vulnerable to pathogens. Malakoplakia could be a response to the chronic inflammation that further led to the development of the hepatic abscess. This is the first reported case in which pancreatic heterotopia caused a liver abscess. The rare combination of both entities has most likely led to the clinical presentation of an intrahepatic abscess mimicking an intrahepatic cholangiocarcinoma. Pancreas heterotopia can mimic both intrahepatic cholangiocarcinoma and Klatskin tumors [5].

The exact incidence of pancreatic heterotopia is ultimately unclear. If one follows the incidence reported by Terada et al. [2] it may be more common than expected. Keeping in mind that the genesis of 50 % of liver abscesses remains unclear [10], pancreatic heterotopia might be an underreported reason for formation of otherwise unexplained liver abscess formations.
